# Study on the Curative Effect and Safety of Radiofrequency Catheter Ablation of Paroxysmal Atrial Fibrillation via Zero-Fluoroscopy Transseptal Puncture under the Dual Guidance of Electroanatomical Mapping and Intracardiac Echocardiography

**DOI:** 10.1155/2021/5561574

**Published:** 2021-05-24

**Authors:** Fei Hang, Liting Cheng, Zhuo Liang, Ruiqing Dong, Xinlu Wang, Ziyu Wang, Zefeng Wang, Yongquan Wu

**Affiliations:** ^1^Department of Cardiology, Beijing Anzhen Hospital, Capital Medical University, Beijing, China; ^2^Affiliated Hangzhou First People's Hospital, Zhejiang University School of Medicine, Hangzhou, China

## Abstract

**Aims:**

3D electroanatomical mapping combined with intracardiac echocardiography- (EAM-ICE-) guided transseptal puncture has been proven safe and effective during the radiofrequency catheter ablation (RFCA) procedure used to treat paroxysmal atrial fibrillation (PAF). In this study, we aimed to compare the curative effect and safety of RFCA via F (fluoroscopy) and zero-fluoroscopy transseptal puncture guided by EAM-ICE in patients with PAF.

**Methods and Results:**

A prospective study in which 110 patients with PAF were included and assigned to two groups was conducted. Fifty-five (50%) patients were enrolled in the EAM-ICE group, whereas the other 55 (50%) patients were enrolled in the F group. There were no significant differences in baseline characteristics between the two groups. The transseptal duration time was longer in the EAM-ICE group (19.8 ± 3.0 min vs. 8.6 ± 1.2 min, *p* ≤ 0.01); however, fluoroscopy was not used in the EAM-ICE group compared with the *F* group (0 mGy vs. 109.1 ± 57.9 mGy). Similarly, there was also no significant difference in the recurrence rate of atrial fibrillation between the EAM-ICE and *F* groups (25.5% vs. 18.2%, *p*=0.356).

**Conclusion:**

RFCA via EAM-ICE-guided zero-fluoroscopy transseptal puncture in patients with PAF is safe and effective for long-term follow-up.

## 1. Introduction

Zero-fluoroscopy catheter ablation for ventricular tachycardia and supraventricular tachycardia has been proven effective and practicable in routine clinical practice, especially for children and pregnant women [1, 2]. However, atrial fibrillation (AF) ablation without fluoroscopy guidance seems relatively complex due to transseptal puncture. The rigid anatomical structure of the foramen ovale, adjacent to the ascending aorta and posterior wall of the right atrium, creates difficulty in transseptal puncture [3].

Trials related to both intracardiac echocardiography (ICE) and transesophageal echocardiography- (TEE-) guided transseptal puncture have proven to be feasible [4, 5]. However, there are still several limitations. ICE and TEE provide only 2D visualization and cannot directly reflect the three-dimensional anatomical structure of atrial and adjacent tissues. Therefore, a novel approach is needed to solve this problem.

In our previous study, we performed transseptal puncture guided by ICE combined with 3D electroanatomical mapping systems (EAMs) during the ablation procedure of AF [6]. The acute outcomes were gratifying. However, long-term complications and AF recurrence following this novel approach still need to be evaluated. In this clinical study, related outcomes will be evaluated.

## 2. Materials and Methods

### 2.1. Study Design and Study Population

We conducted a prospective, cohort study from April 2019 to September 2020. This clinical study was approved by the Beijing Anzhen Hospital Medical Ethics Committee. Patients who were enrolled in this study provided written informed consent.

A total of 110 consecutive patients at Beijing Anzhen Hospital with paroxysmal atrial fibrillation (PAF) were enrolled in this study. They were divided into two groups (EAM-ICE group and F group) according to the method of transseptal puncture. The inclusion criteria included (a) adults 18 to 80 years old; (b) use of 12-lead electrocardiography or Holter monitoring to diagnose PAF, which is defined as spontaneous termination of AF or AF onset <7 days before catheter ablation; and (c) symptomatic PAF patients amenable to RFCA treatment or refractory or intolerant to antiarrhythmic medication.

The exclusion criteria were as follows: (a) left atrial anteroposterior diameter >50 mm; (b) left atrial appendage thrombosis; (c) abnormal cardiac structure disease (i.e., severe aortic, tricuspid, or mitral malformations; tetralogy of Fallot; ventricular and atrial septal defects); (d) mental disorder; (e) history of cryoballoon ablation, RFCA, and/or cardiac surgery; (f) septic shock; (g) eGFR <30 ml/min; (h) pregnancy; (i) advanced malignant tumor; (j) heart failure; and (k) major hydropericardium or cardiac tamponade.

### 2.2. Procedure Characteristics

#### 2.2.1. F-Guided Catheter Ablation

**Figure 1:**
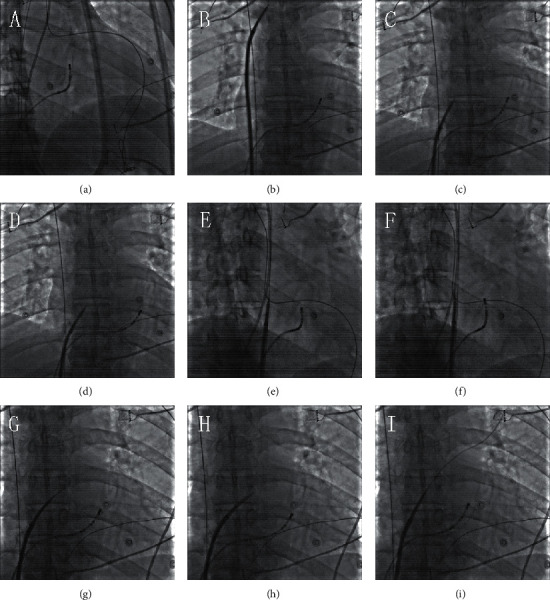
Transseptal puncture with fluoroscopy. (a) At the right anterior oblique (RAO) angle of 30°, a 10-polar diagnostic catheter was placed in the coronary sinus. (b) At the anterior posterior (AP) position, the long sheath was sent into the superior vena cava (SVC) through the guidewire. Then, we removed the guidewire and sent a transseptal needle into the long sheath. The distance between the tip of the long sheath and the tip of transseptal needle was about 1 cm. (c, d) Upon withdrawal of the long sheath and the transseptal needle together, the tip of the sheath was observed to jump twice. After jumping for the second time, the tip was pointing toward to the foramen ovale. (e) RAO 45° was used to confirm that the tip of the sheath was vertical to the atrial septum. (f) A transseptal needle was used to puncture the atrial septum. (g) Contrast medium was injected into LA through the needle, and a dark thick line was shown under fluoroscopy at the AP position. (h, i) We pushed dilator inside the long sheath through the transseptal needle into the LA. Then, the needle was withdrawn, and the long wire was placed in the left superior pulmonary vein.

**Figure 2:**
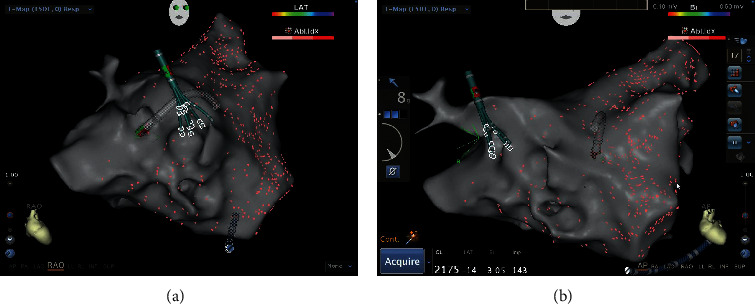
Establishing the left atrium model with a PentaRay catheter in a patient in the F group.

**Figure 3:**
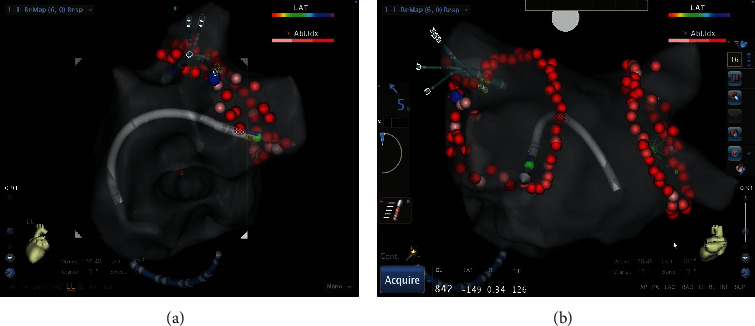
Radiofrequency catheter ablation of paroxysmal atrial fibrillation accomplished in the F group.

After bilateral femoral venous puncture, two guidewires and 6-F introducer sheathes (Input; Medtronic, Minneapolis, USA) were inserted. Under the guidance of fluoroscopy, a 10-polar diagnostic catheter was placed in the coronary sinus (CS). Patients in the F group underwent transseptal puncture guided by fluoroscopy (details are described in Figure 1). Transseptal puncture was performed twice, and then a multipolar catheter (PentaRay; Biosense Webster, Diamond Bar, CA, USA) and an ablation catheter were inserted into the left atrium (LA). Left atrial mapping was directed by PentaRay (Figure 2). Circumferential pulmonary vein isolation (CPVI) was performed using an ablation catheter (Thermocool Smarttouch; Biosense Webster) (Figure 3). Power control mode at 35 W (irrigation flow 17 mL/min) was used during CPVI. During the procedure, we use Visitag (Carto, Biosense Webster) to guide the performance of contact force (CF; >3 g for 25% of time) and catheter stability (2.5 mm for 3 seconds). The ablation index (AI) targeted at the anterior wall of LA was 500, and that targeted at the posterior wall was 400. The activated clotting time (ACT) was maintained between 300 and 350 s by intravenous heparin.

#### 2.2.2. EAM-ICE-Guided Catheter Ablation

**Figure 4:**
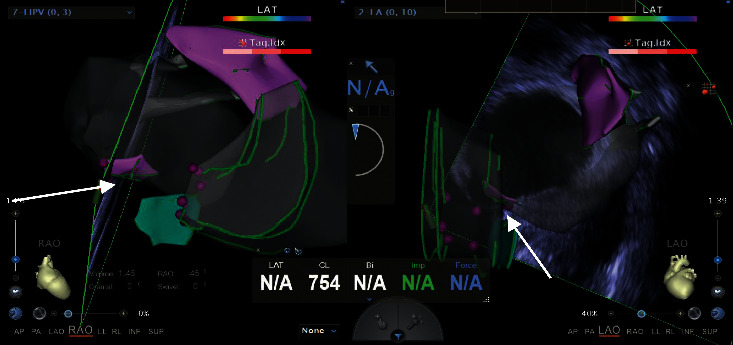
Establishing the left atrium model with intracardiac echocardiography (ICE) and using electroanatomical mapping systems (EAMs) and ICE to confirm the location of the foramen ovale (shown by the arrow).

**Figure 5:**
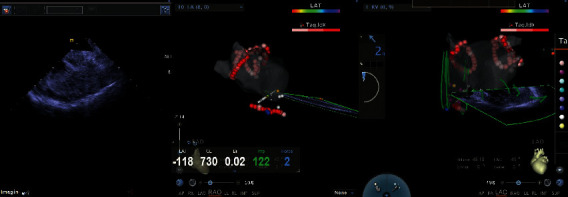
Radiofrequency catheter ablation of paroxysmal atrial fibrillation accomplished via zero-fluoroscopy transseptal puncture guided by EAM-ICE.

Details of the related procedure were described in our previous publication [6]. The process of using EAM-ICE to establish left atrium model is shown in Figure 4.  Figure 5 shows the use of EAM-ICE guidance to complete the ablation of PAF.

### 2.3. Baseline and Follow-Up Evaluation

Patients' baseline characteristics such as age, sex, and past history body mass index (BMI) were recorded. The findings of cchocardiography findings (i.e., LA anteroposterior diameter and left ventricular ejection fraction (LVEF)) were evaluated. The recurrence rate at 6 months was also measured (recurrence was defined as AF lasting for more than 30 s within 6 months after CPVI).

### 2.4. Randomization, Treatment Grouping, and Study Outcomes

Patients who met the inclusion criteria but not the exclusion criteria were assigned to the F group or EAM-ICE group. The EAM-ICE group underwent transseptal puncture guided by ICE (Soundstar; Biosense Webster, Diamond Bar, CA, USA) and a 3D EAM system (Carto; Biosense Webster, Diamond Bar, CA, USA). The F group underwent transseptal puncture guided by fluoroscopy only. The primary outcome of this study was the safety of the related procedures, which were major complication rates (i.e., cardiac perforation, acute myocardial infarction, hydropericardium, atrial esophageal fistula, malignant arrhythmia, and sudden cardiac death). The secondary outcome was the recurrence rate at 6 months during follow-up.

### 2.5. Statistical Analysis

SPSS statistical software (IBM, version 23) was performed in data analysis. Normally distributed continuous variables were expressed as the means ± standard deviations, whereas nonnormally distributed data were expressed as medians (Q1, Q3). Independent samples *t*-test was used compare variables between groups for normally distributed data, and the comparison between groups for nonnormally distributed data was performed by Mann–Whitney *U*-test. *p* < 0.05 was considered statistically significant.

## 3. Results

### 3.1. Baseline Clinical Characteristics

110 patients with PAF were included in this study, 55 (50%) of whom were female. 55 (50%) patients were enrolled in the EAM-ICE group. The patients' detailed baseline clinical characteristics are shown in Table 1. There were no statistically significant differences in baseline characteristics between the two groups.

### 3.2. Outcomes

All patients underwent successful transseptal puncture (110/110, 100%). No procedural complications (i.e., cardiac perforation, hydropericardium, malignant arrhythmia, sudden cardiac death, atrial esophageal fistula, or acute myocardial infarction) occurred in either group. We evaluated the total procedure time and transseptal duration in both groups. The total procedure duration was defined as the duration of the patient's stay in the operating room. Transseptal duration was defined as the time from when the long sheath was inserted into the SVC until the long sheath was inserted into the LA. The transseptal duration was longer in the EAM-ICE group (12.8 ± 3.0 min vs. 109.1 ± 57.9 min, *p* ≤ 0.01); however, there was no use of fluoroscopy in the EAM-ICE group (0 mGy vs. 109.1 ± 57.9 mGy) (Table 2). Additionally, there was no significant difference in the recurrence rate between the EAM-ICE and F groups (25.5% vs. 18.2%, *p*=0.356).

## 4. Discussion

Considering our previous study together with the current study, we deem zero-fluoroscopy transseptal puncture guided by ICE and 3D EAM system to be safe and effective [6]. In our previous study, we described the procedure of EAM-ICE-guided transseptal puncture in detail and concluded that the procedure is feasible. However, the safety and efficacy of this procedure for AF ablation remains to be estimated. In the current study, we compared the safety and efficacy of catheter ablation of PAF by using EAM-ICE and F to perform transseptal puncture. No procedural complications occurred in either group. The transseptal procedure and total procedure may take longer for the EAM-ICE group patients, which is due to the relatively complex operation, including the mapping of the foramen ovale and insertion of the ICE catheter.

Fluoroscopy is the foundation of electrophysiology and pacing; however, it does lead to health problems for both patients and physicians. Zero-fluoroscopy procedures have been conducted in recent years. Under the guidance of ultrasound, transseptal puncture and mapping can be conducted. However, ultrasound provides only 2D visualization, which restricts its usage. In contrast, 3D mapping systems have been universally used for catheter ablation [7–9]. Under the direction of EAM, the tip of the transseptal needle can be visualized. Furthermore, due to the specific anatomical features of the foramen ovale, it has low-voltage reading electrography yields, which are prominent in the use of EAM [10]. Yu et al. [11] conducted total 3D transseptal puncture (T3D) in 276 patients without the guidance of ICE or TEE. Related trials have proven that catheter ablation can be performed without fluoroscopic guidance [12]. In our previous study, we performed transseptal puncture under the guidance of ICE combined with EAM and proved that this method is safe and effective. However, long-term complications and AF recurrence following PAF ablation via this new transseptal method still need to be evaluated. In this clinical study, we evaluated related outcomes. There was no significant difference in the recurrence rate between the EAM-ICE and F groups (25.5% vs. 18.2%, *p*=0.356). Additionally, the total procedure duration and transseptal duration were longer in the EAM-ICE group (166.3 ± 23.9 min vs. 140.4 ± 30.8 min, *p*=0.015 and 19.8 ± 3.0 min vs. 8.6 ± 1.2 min, *p* < 0.001, respectively), which may be related to the more complex procedure in the EAM-ICE group. However, there was no use of fluoroscopy in the EAM-ICE group (0 mGy vs. 109.1 ± 57.9 mGy). This study added solid evidence that EAM-ICE is safe and efficacious.

In this study, we found that the total procedure duration and transseptal duration were longer in the EAM-ICE group than in the F group. We consider that there were several reasons for this issue. First, it took time to place the ICE catheter into the right atrium and find a suitable ultrasound view that could satisfactorily show the foramen ovale. Furthermore, it also took time to map low-voltage areas with the ablation catheter. As the operator's experience improves, the time is expected to be shortened.

In this study, we also found that it is safe to perform PAF ablation via zero-fluoroscopy transseptal puncture guided by EAM-ICE during short- and long-term follow-up, which is in accordance with our previous study. We think there were several reasons for this. First, in our study, we used EAM and ICE to confirm the location of the foramen ovale, which could increase the safety of transseptal puncture and reduce the risk of related complications such as pericardial tamponade. Second, we used ICE to build a model of the left atrial appendage and esophagus in this study, which is beneficial for reducing the risk of left atrial appendage perforation and esophageal injury during the ablation procedure. Third, in this study, ICE clearly showed the junction structure between the left atrium and pulmonary veins, which could improve the accuracy of ablation and reduce the risk of stenosis of pulmonary veins after ablation. Furthermore, in our study, each patient in the EAM-ICE group underwent only one transseptal puncture, which could also lead to a lower incidence of complications.

There were no differences in the recurrence incidence of AF between the two groups, although mapping catheters such as a PentaRay or Lasso were not used in the EAM-ICE group. There may be several reasons for this. First, we can effectively build a model of the left atrium by using ICE, which is merged into the Carto system. Second, in our center, the operator is experienced in performing the procedure and does not rely on mapping catheters. Third, we can use ICE to clearly indicate the junction of the pulmonary veins and left atrium, which is helpful for improving the accuracy of ablation.

In general, RFCA via zero-fluoroscopy transseptal puncture is safe and effective. At the same time, because the atrial septum was punctured only once and mapping catheters were not used, it seems cost-effective. We look forward to more follow-up studies involving more patients on this issue.

## 5. Limitations

We have not performed this trial in a random fashion, which limits the strength of the conclusions. The number of patients who were included in this study was relatively small, and the follow-up time was relatively short, which is another limitation of this study.

## 6. Conclusion

It is safe and effective to perform the EAM-ICE procedure for catheter ablation of atrial fibrillation. Thus, the use of a zero-fluoroscopy approach for PAF is feasible.

## Figures and Tables

**Table 1:** Baseline patient characteristics.

	EAM-ICE (*n* = 55)	*F* (*n* = 55)	*p* value
Age, y	59.7 ± 8.7	58.5 ± 10.0	0.524
Male, *n* (%)	39(70.9%)	38 (69.1%)	0.835
BMI, kg/m^2^	25.9 ± 2.6	25.3 ± 3.2	0.297
AF duration, months	24 (7, 36)	24 (6, 36)	0.618
LDL, mmol/L	2.5 ± 1.2	2.6 ± 1.2	0.571
AST, U/L	25.3 ± 12.3	24.8 ± 14.6	0.844
Creatinine, *μ*mol/L	73.5 ± 14.8	70.4 ± 13.7	0.249
Hypertension, *n* (%)	28 (50.9%)	22 (40%)	0.251
Diabetes, *n* (%)	11 (20%)	10 (18.2%)	0.808
CHD, *n* (%)	6 (10.9%)	12 (21.8%)	0.122
Smoking, *n* (%)	15 (27.3%)	17 (30.9%)	0.279
CHA_2_DS_2_-VAS_C_≥2, *n*(%)	25 (45.5%)	25 (45.5%)	1.000
LA AP diameter, mm	38.4 ± 5.2	37.8 ± 5.0	0.563
EF, %	63.8 ± 4.7	61.5 ± 7.7	0.069

BMI, body mass index; AF, atrial fibrillation; LDL, low-density lipoprotein; AST, aspartate aminotransferase; CHD, coronary heart disease; LA, left atrial; AP diameter, anteroposterior diameter; EF, ejection fraction.

**Table 2:** Outcomes.

	EAM-ICE (*n* = 55)	*F* (*n* = 55)	*p* value
Major complication (%)	0	0	1.000
Total procedure duration^a^, min	166.3 ± 23.9	140.4 ± 30.8	0.015
Transseptal duration^b^, min	19.8 ± 3.0	8.6 ± 1.2	≤0.01
Radiation dosage, mGy	0	109.1 ± 57.9	≤0.01
Recurrence (5)	14 (25.5%)	10 (18.2%)	0.356

^a^Total procedure duration was defined as the duration of the patient's stay in the operating room. ^b^In the EAM-ICE group, transseptal duration was defined as the time beginning when the long sheath was inserted into the SVC until the long sheath was inserted into the LA; in the F group, transseptal duration was defined as the time beginning when the first long sheath was inserted into the SVC until the second long sheath was inserted into the LA.

## Data Availability

The data used to support the findings of this study are included within the article.
